# Student Empowerment and Graduate Competency Development in Higher Education: Evidence from an Independent Campus Programme

**DOI:** 10.12688/f1000research.179643.2

**Published:** 2026-06-25

**Authors:** Indra Prasetia, Tutik Sugesti, Nurmadiah Nurmadiah, Bambang Panca Syahputra, Arief Aulia Rahman, Edy Suprayetno, Gusman Lesmana

**Affiliations:** 1 Postgraduate, Universitas Muhammadiyah Sumatera Utara, Medan, North Sumatra, 20371, Indonesia; 2 Postgraduate, Universitas Muhammadiyah Sumatera Utara, Medan, North Sumatra, 20371, Indonesia; 3 Postgraduate, Universitas Muhammadiyah Sumatera Utara, Medan, North Sumatra, 20371, Indonesia; 4 Faculty of Teacher Training and Education, Universitas Muhammadiyah Sumatera Utara, Medan, North Sumatra, 20238, Indonesia; 5 Faculty of Teacher Training and Education, Universitas Muhammadiyah Sumatera Utara, Medan, North Sumatra, 20238, Indonesia; 6 Faculty of Teacher Training and Education, Universitas Muhammadiyah Sumatera Utara, Medan, North Sumatra, 20238, Indonesia; 7 Faculty of Teacher Training and Education, Universitas Muhammadiyah Sumatera Utara, Medan, North Sumatra, 20238, Indonesia

**Keywords:** independent campus, student empowering, graduate competence

## Abstract

**Background:**

The independent campus programme is an educational initiative that offers students the opportunity to study more flexibly through a variety of learning activities outside their degree programmes and off-campus. However, the role of student empowerment in this programme has not yet been explored in depth. This study aims to conduct an in-depth exploration of how students are empowered in the independent campus programme and the impact of this empowerment on the development of graduates’ competencies and their readiness for the workforce.

**Methods:**

This study employs a descriptive qualitative approach to explore student empowerment and the competencies and skills acquired by graduates from the independent campus program. Data were collected through semi-structured interviews with 17 respondents selected through purposive sampling, and the data were analyzed using thematic analysis, which involves data grouping, pattern identification, theme formation, and interpretation or drawing of conclusions.

**Result:**

This study reveals two main findings. First, student empowerment in the independent campus program is achieved through six approaches: facilitating, streamlining, consulting, collaborating, mentoring, and supporting. Second, the indenpendent campus program contributes to the development of students’ competencies and skills, namely: leadership, teaching skills, communication skills, problem-solving, critical thinking, analytical skills, networking, and technical skills. The independent campus program provides students with the opportunity to gain hands-on work experience, enabling them to develop skills relevant to current and future workplace needs.

**Conclusions:**

Independent campus program make a positive contribution to the development of graduates’ competencies at universities. Through flexible, real-world, practice-based learning experiences, students are encouraged to become more active, independent, and directly engaged in the learning process, thereby strengthening their readiness to meet the demands of the workplace.

## 1. Introduction

Higher education in Indonesia continues to make continuous improvements to enhance the quality of education in the face of global challenges. Therefore, universities are required to be innovative through the application of more flexible national standards, so that they can develop into independent, excellent institutions capable of implementing various innovative educational programmes. ^1^ One of the educational programs currently being implemented at universities is the independent campus. This programme was launched by the Minister of Education and Culture of the Republic of Indonesia in 2020, this programme aims to provide students with the opportunity to master various fields of study and relevant learning experiences, so that they are better prepared for the world of work. ^2, 3^ Independent campus is essentially an education system that prioritises flexible, student-centred learning that is relevant to the needs of the workplace. ^4^ Under the educational concept of the independent campus programme, students are given the opportunity to study in a more flexible manner, gain hands-on experience outside the campus, and develop skills relevant to the professional world. ^5^

The independent campus is an educational programme designed to adapt to the era of globalisation driven by industry needs, as it provides students with the opportunity to gain hands-on learning experience in the workplace, develop practical skills, and enhance their ability to adapt to technological advancements and industry requirements. ^6^ Through internships, research projects and entrepreneurship initiatives, students are expected to gain a deeper understanding of the real-world needs of the workplace. ^7^ These experiences not only broaden their practical knowledge but also help students develop professional skills relevant to their field. ^8^ The independent campus programme is not solely focused on academic development; it also plays a key role in developing students’ character and soft skills, such as interpersonal skills, leadership, creativity and innovation, which are highly relevant in today’s dynamic workplace. ^3, 9, 10^ Furthermore, teaching within the independent campus programme places greater emphasis on student-centred learning, experience-based learning, and the principle of alignment between higher education and the needs of the workplace. ^4, 11^

Essentially, the independent campus programme offers a range of benefits for students, particularly in enhancing their learning experience, practical skills and readiness for the world of work. ^12^ However, the benefits of the independent campus programme have not yet been fully matched by student participation, which remains relatively low to date. ^13^ National data for the 2020–2024 period shows that the number of students involved was recorded at around 725,000. Meanwhile, in 2024, only around 193,000 students were enrolled in this programme out of a total of around 8.2 million students across Indonesia. Furthermore, of the approximately 4,523 higher education institutions in Indonesia, only around 1,300 have implemented the programme. ^13, 14^ This situation indicates that student participation and the readiness of higher education institutions for the programme remain low.

In addition to challenges regarding participation, the independent campus programme still faces issues relating to student empowerment. ^15^ At some universities, students have not yet been given sufficient opportunity to take the initiative and play an active role in programme activities or work placements, they are often limited to routine administrative tasks that do not support the development of their skills, creativity and independence. ^16, 17^ The placement of students in roles that do not always align with their interests and fields of study also means that the development of their academic skills is less than optimal. ^18^ There are still rigid and inflexible regulations, as well as bureaucratic structures at a number of universities, which are hindering the exploitation of the opportunities offered by the independent campus programme. ^19^ Some lecturers are still accustomed to traditional teaching methods and are finding it difficult to adapt to the rapid changes brought about by the policy. ^20^ Student empowerment, the learning experience and academic support, as well as the readiness of higher education institutions, are key factors in the success of this programme. ^16^ Empowerment and learning support within the independent campus programme are vital to ensuring that students can gain meaningful learning experiences that are relevant to the needs of the workplace. ^15, 18, 21^ Empowering students in the independent campus programme provides them with opportunities to take the initiative, participate actively, and develop critical thinking skills, creativity and independence through a range of out-of-class learning activities, such as work placements, research projects or community engagement. ^20, 22^ Academic support from universities, in the form of staff supervision, flexible academic systems and collaboration with industry partners, is a key factor in enabling students to make the most of the independent campus programme. ^23^ With adequate empowerment and support, students not only gain practical experience but are also able to develop the professional skills that will prepare them for the world of work. ^21– 23^

The independent campus programme remains one of the key strategies for higher education institutions in Indonesia in improving the quality of their graduates. ^24^ Therefore, this programme needs to be reviewed and evaluated to ensure its effectiveness in supporting the development of students’ skills, their readiness to enter the world of work. ^25, 26^ A number of surveys have shown that students participating in the independent campus programme benefit from improved practical skills, real-world work experience and the ability to adapt to the world of industry. According to a survey conducted by the Indonesian government of 824 university leaders, 14,869 lecturers and 124,562 students at autonomous universities, virtually all university leaders (99%), lecturers (98%) and students (98%) consider the independent campus programme to be beneficial and to have a positive impact on graduates. ^13, 27^ Surveys conducted by several universities also show that students participating in the independent campus programme find it easier to secure employment, taking just 7–8 months after graduation; the independent campus programme effectively supports students in entering the workforce immediately after graduation; and 33% of students from economically disadvantaged backgrounds take part in internship and independent study programmes. ^20, 28, 29^

Although various studies have provided an overview of the impact and benefits of the independent campus programme, existing research is still limited to satisfaction surveys regarding the programme’s implementation or general impact. Through this research, we aim to build upon the findings of previous studies by conducting an in-depth examination of student empowerment within the independent campus programme and its impact on the development of graduates’ competencies and readiness to meet the demands of the workplace. This will provide new insights and knowledge for higher education institutions, highlighting that student empowerment is a key factor in determining the extent to which graduates acquire the skills and readiness required to enter the workforce. This study also aims to establish a relationship between student empowerment and the development of student competencies, as well as the impact of this on graduates’ readiness for the world of work, thereby contributing to the development of a theory or model of the relationship between student empowerment and its impact within the context of the independent campus programme in higher education institutions.

## 2. Literature review

### 2.1 The independent campus programme

The independent campus initiative is a higher education policy that provides students with the opportunity to study in a more flexible and innovative manner, tailored to the needs of the workplace, through a range of activities both outside their degree programmes and beyond the university. ^30^ Conceptually, the independent campus initiative is an approach to higher education that places students at the centre of the learning process by granting them the freedom and flexibility to shape their learning experiences in line with their interests, potential and competence development needs. ^31, 32^ This concept emphasises learning that takes place not only within the classroom, but also through a range of activities outside the university that are relevant to the world of work and the needs of society. ^5^ This concept emphasises learning that takes place not only within the classroom, but also through a range of activities outside the university that are relevant to the world of work and the needs of society. ^17^ Within the framework of the Indonesian Government’s policy, independent campus is synonymous with “independent learning, independent campus” which is understood as an initiative to transform higher education by fostering more innovative, adaptive and experiential learning processes. ^33– 35^ Through this concept, students are encouraged to develop academic competencies, professional skills, and the ability to think critically, creatively, collaboratively. ^6^

Independent campus off ers unprecedented academic freedom in the higher education system. ^36^ The programme gives students the flexibility to gain learning experiences within and beyond their study programme, covering a wide range of activities, such as: (1) student exchange, (2) internship or practical work, (3) campus teaching, (4) research, (5) humanitarian projects, (6) entrepreneurial activities, (7) independent projects, (8) village building or real work college, (9) state defence. ^9, 13, 16^ The independent campus is based on the idea of humanism theory. ^37^ Humanistic education is that students are placed as free and independent subjects to determine the choices and direction of their lives. ^38^ Humanist education emphasises communication and personal relationships based on an atmosphere of love, understanding, and the need for mutual respect. Humans as a single figure and have characteristics that are different from other individuals, therefore each individual is fully concentrated on himself. ^39^ Humanistic education theory stems from three major philosophical theories: pragmatism, progressivism and existentialism. Pragmatism believes that education should be democratised so that everyone can be involved in the education. ^37, 40^ Progressivism emphasises the freedom of self-actualisation for all learners. Meanwhile, in the view of existentialism, individual uniqueness is the main aspect that must be considered to realise humanist education. When viewed from these three basic teri, students have full responsibility for their own lives. ^41^

### 2.2 Implementation of independent campus

The “independent campus” initiative is a policy of the Government of the Republic of Indonesia, implemented through the Ministry of Education, aimed at encouraging students to master various fields of knowledge that are useful for entering the workforce. ^10^ Education and learning at the independent campus emphasize the concept of deep learning in a more authentic community setting. ^42^ Independent campuses give students the opportunity to choose the courses they want to take. ^43^ The independent campus program is a higher education initiative that is more flexible, innovative, and tailored to students’ needs. This approach allows students to choose courses and programs that align with their interests. ^5^ The independent campus offers educational programs such as student exchanges, internships or work placements, teaching assistantships in academic units, research, entrepreneurship programs, humanitarian projects, independent study, village development, or field studies. ^13^ Independent campuses aim to encourage students to gain learning experience with a variety of additional competencies. in the study programme or off campus. ^44^ The purpose of implementing an independent campus is so that students will have the ability to master various sciences that are useful in the world of work. ^25^ The objectives of the independent campus programme include improving graduates’ competencies to be better prepared for the challenges of the world of work, providing contextualised hands-on learning experiences, and encouraging innovation and creativity through practical projects. ^31^

Implementing a independent campus requires effective collaboration among various stakeholders, ranging from the university to students and partners. ^3^ Just as the higher education system must guarantee students’ rights as set forth in Regulation No. 3 of 2020 on National Standards for Higher Education, namely (1) can take credits outside the university for a maximum of 2 semesters or the equivalent of 40 credits, (2) can take credits in different study programmes in the same university for 1 semester or the equivalent of 20 credits, (3) compile policies or academic guidelines to facilitate learning activities outside the study programme, (4) create cooperation documents with partners. ^30, 45^ The role of the faculty in the independent campus programme is: (1) to draw up a list of faculty-level courses that can be taken by students from various degree programmes, (2) to draw up cooperation agreements with relevant partners. ^46^ The role of the degree programme includes (1) to develop or adapt the curriculum to the implementation model, (2) to facilitate students wishing to undertake cross-programme study within the university, (3) to offer courses that may be taken by students outside their degree programme and outside the university, along with the relevant requirements, (4) recognising the equivalence of courses with learning activities outside the degree programme and outside the university, (5) if there are courses not yet fulfilled through learning activities outside the degree programme and outside the university, then alternative online courses shall be provided. ^34, 43^ The roles of students in the implementation of a independent campus are (1) consult with an academic advisor regarding courses or programmes to be taken outside the degree programme, (2) register for activities outside the degree programme, (3) meet the requirements for activities outside the degree programme, (4) including participating in the selection process where applicable, (5) participate in activities outside the degree programme in accordance with the provisions of the applicable academic guidelines. ^13^ Furthermore, no less important is the role of partners, namely making cooperation documents and implementing the programme of activities outside the study programme in accordance with the provisions in the cooperation document. ^6^

### 2.3 Effectiveness of the independent campus programme: Student empowerment and developing graduate competencies

Effectiveness is something that shows the level of achievement of a goal. An effort can be said to be effective if it achieves its ideal goal. ^47, 48^ The effectiveness of the independent campus programme is the success in achieving the expected goals in the form of broad student knowledge and skills and graduates who are competitive in the world of work. ^3, 17^ The effectiveness of an independent campus through a process that provides contextualised field opportunities that strengthen students’ overall capabilities, prepare them for employment, or build new careers. ^31^ One indication of the success of independent learning is being able to develop the competence and competitiveness of graduates. ^7^ The quality of graduates who meet the demands expected by users or the world of work. ^49^ Measures of graduate capability can be seen through the competent use of knowledge, skills and values that are aligned with work needs, fulfil user demands and contribute to the achievement of institutional goals. ^50, 51^

Competitive higher education graduates are graduates who have various types of abilities following stakeholder needs. ^40^ Graduates who are able to think critically, explore, experiment, have integrity and utilise their skills. ^46^ Explained that the quality factors of competitive graduates are identified with communication skills, organisational skills, leadership, team skills, ethics and cooperation. ^52^ Research on the impact of independent campuses shows that participants in independent campus programmes have a three-month shorter job waiting time after graduation, and they also have an average salary 2,2 times greater than the national average. ^16^

When linked to community development strategies, the independent campus programme is a form of empowerment, namely freedom and autonomy for educational institutions, students and lecturers. ^27^ Universities make policies that support and facilitate, while lecturers are not shackled by excessive rules, while students can choose the field of study that suits their interests. ^53^ Student empowerment is a process or effort that aims to increase the capacity, independence, and active role of students in various aspects of life, both on campus and in society. This empowerment involves developing competence, increasing understanding, practical experience, and building positive attitudes and values in students. ^54^ The purpose of student empowerment is to prepare them as competent, competitive individuals who have a role in building society. ^55^ Through empowerment, students can optimise their potential, develop skills, knowledge and broaden their horizons. ^56^ Student empowerment is to provide opportunities for them to grow and develop holistically, so that they can contribute actively and have a positive impact on themselves, universities, society, the nation as a whole. ^53, 54^

Student empowerment on an independent campus is carried out in ways, including: (1) education and learning. Providing relevant, innovative, interactive curriculum, learning that can improve academic skills, work skills, understanding of social, economic, and environmental issues; (2) training and workshops. Organise training and workshops related to skills development such as entrepreneurship, digital skills, creativity, time management, problem solving; (3) practices and internships. Provide opportunities for students to take part in internships or work practices in industries or organisations related to their field of study. This helps them gain real-world experience, develop practical skills, and expand their professional network; (4) community service. Invite students to engage in community service activities, such as social programmes, community building, or social entrepreneurship initiatives; (5) guidance and counselling. Provides support and guidance to students in developing personal potential, setting career goals, overcoming challenges, building physical and mental well-being. ^31, 53, 54^

Empowerment is interpreted as a goal and process. ^57^ As a goal, empowerment is a state to be achieved, namely students who have strength or power and empowerment that leads to independence according to the types of power. Empowerment as a process is: (1) enabling is creating an atmosphere or climate that allows student potential to develop optimally, (2) empowering is strengthening students’ knowledge and abilities, (3) supporting is providing guidance and support to students so that they are able to carry out their roles and functions, (4) fostering is maintaining conducive conditions so that there remains a balance of power distribution between universities, lecturers and students. ^31, 52, 57^

The independent campus programme is essentially a scheme that provides students with the opportunity to develop their innovation, creativity, skills, character and independence in seeking and acquiring knowledge through real-world experiences and on-the-ground dynamics. ^20^ The independent campus programme is believed to be capable of producing graduates with both technical and non-technical skills. One example is the practical internship programme. This practical internship programme provides students with hands-on experience, as learning takes place directly in the workplace. ^35^ In addition to mastering technical skills, students also develop problem-solving and analytical skills, as well as soft skills such as work ethic, communication and teamwork. Independent universities have proven successful in enhancing students’ competencies after graduation. ^8^ This is reflected in the increase in critical thinking, creativity, communication, problem-solving skills gained by students through learning experiences outside the scope of their classroom. ^5^

The implementation of the independent campus model aims to prepare students to become resilient, adaptable and responsive individuals who can meet the demands of the modern world, and who are ready to become future leaders with a strong sense of national pride. ^58^ Opening up a wide range of opportunities for students to broaden, deepen and enhance their knowledge, skills in the real world, in line with their potential, talents, interests, enthusiasm and aspirations. ^43^ This independent campus develops students’ skills, including (1) critical thinking and decision-making, (2) collaboration and communication, (3) creativity and innovation, (4) networking. ^32, 35, 59^ Producing graduates who are superior, independent, creative and resilient, becoming good learners in accordance with the concept of lifelong learning. ^6^ The benefits of the independent campus programme for the development of students’ abilities and skills include (1) practical skills, where students gain hands-on experience in the world of work through internships and projects; (2) professional networks, where they expand their connections with industry and professionals; (3) entrepreneurship, where they help develop their business and entrepreneurial spirit; (4) soft skills, where they improve their communication, collaboration, and self-management skills; (5) cross-disciplinary experience, where they enrich their horizons by studying outside their study programme. ^25, 43^ The research findings show that independent campuses are able to produce a graduate profile that is more competitive in the labour market. They have more relevant experience and skills as well as the ability to adapt quickly to changes in the work environment. ^10, 36, 44^

## 3. Research methods

### 3.1 Research design

This study is a descriptive qualitative study, which aims to describe or depict phenomena as they occur naturally and in depth, without intervening in or manipulating the variables under investigation. A descriptive qualitative approach is an approach that describes reality or events as they are, based on information from informants or the situation under study. ^60– 62^ Research that seeks to understand the researcher’s experiences or perspectives, and to interpret data in order to identify patterns or themes. ^63^ A descriptive qualitative approach was employed in this study to explore various forms of student empowerment, the implementation process of the independent campus programme, and its contribution to the development of graduate competencies in higher education institutions, based on the experiences and perceptions of the participants. This study is based on a constructivist-interpretative paradigm, which holds that social reality is shaped through individuals’ experiences and interactions and is understood through the participants’ interpretations of meaning. Consequently, this study aims to explore and interpret the participants’ experiences, perceptions and understanding in order to gain an in-depth understanding of the contribution of the independent campus programme to the development of graduates’ competencies.

### 3.2 Participant

The research participants were determined by purposive sampling, namely 17 participants consisting of 9 students, 5 lecturers and 3 graduates from state universities in Medan city, North Sumatra Provincy, Indonesia. While the criteria for determining student participants, namely (1) active students in higher education, (2) have completed ≥5 semesters of study, (3) participating in an independent learning programme for 2 semesters off their campus. The criteria for graduates are (1) having completed studies in higher education, (2) having experience in independent campus programmes and having worked ≥2 year. Lecturers are supervisors and companions of students in independent learning programmes, have a Doctoral degree and have expertise in their fields. Purposive sampling is a sampling technique with certain considerations. ^64, 65^ The reason for using purposive sampling technique is because it is suitable for qualitative research, or studies that do not generalise. ^61, 62^ Information about the study participants is presented in Table 1.

**Table 1.** Participant information.

Participant	Code	College origin	Gender	Education/Study programme	N
**Student (S)**	S1	College A	Female	Management	5
	S2		Male	Information System	
	S3		Female	Economics and Business	
	S4		Male	Islamic Religious Education	
	S5		Female	Mathematics Education	
	S6	College B	Male	Medical Education	4
	S7		Female	Law	
	S8		Female	Communication Sciences	
	S9		Male	Agriculture	
**Graduate (G)**	G1	College A	Male	Bachelor of Management	2
	G2		Female	Bachelor of Computer	
	G3	College B	Male	Bachelor of Engineering	1
**Lecture (L)**	L1	College A	Female	Doctor of Management	2
	L2		Male	Doctor of Engineering	
	L3	College B	Male	Doctor of Agribusiness	3
	L4		Female	Doctor of Computer Science	
	L5		Female	Doctor of Education	
	N				17

### 3.3 Data collection procedure

The research data was collected through semi-structured interviews, observation and a review of documents. Semi-structured interviews were conducted to gain an in-depth understanding of the informants’ experiences and perceptions regarding student empowerment and the development of graduate competencies in the implementation of the independent campus programme. Meanwhile, observation and documentary research were used to gather additional data to support the findings of the interviews. The observation was carried out to directly monitor the implementation of the independent campus programme, including its delivery, as well as student engagement and empowerment in various learning activities and skills development initiatives. The document study was conducted by examining various relevant documents, such as programme implementation guidelines, activity reports, cooperation agreements, curricula, and data on student and graduate outcomes. This documentary data was used to corroborate and verify the information obtained through interviews and observations, thereby enhancing the credibility of the research findings. The semi-structured interview allows informants to respond broadly, in depth and flexibly to the topics discussed and questions asked. ^61, 62^ In addition to using interviews, research data is also reinforced by data sourced from documents or images, notes or visual recordings available in the field. ^63^

### 3.4 Data analysis

The collected research data was analysed using thematic analysis techniques. Thematic analysis is a method for analysing qualitative data that involves reading through a body of data and searching for patterns of meaning within it in order to identify themes. ^66^ Analysis begins with grouping data, determining patterns, codes, building themes and then interpretation. Then the overall aspects were analysed or interpreted to understand the themes, meanings and relationships between one theme and another which became the focus of the research. ^67, 68^ This thematic analysis focuses on exploring the opinions and experiences of the participants in the implementation of the independent campus, as well as the aspects of empowerment and competency development and the impact of the independent campus. Research data analysis begins with collecting and organising the data, identifying patterns, coding, developing themes, and then interpreting the findings.

## 4. Results

The data for this study was obtained from interviews with 17 respondents regarding the independent campus programme. Data collection focused on three main aspects, namely: (1) programme implementation, (2) the process of student empowerment during the programme, and (3) its impact on the development of graduates’ competencies higher education. The following are the findings from the research themes.

### 4.1 Implementation of independent campus

**Figure 1.**
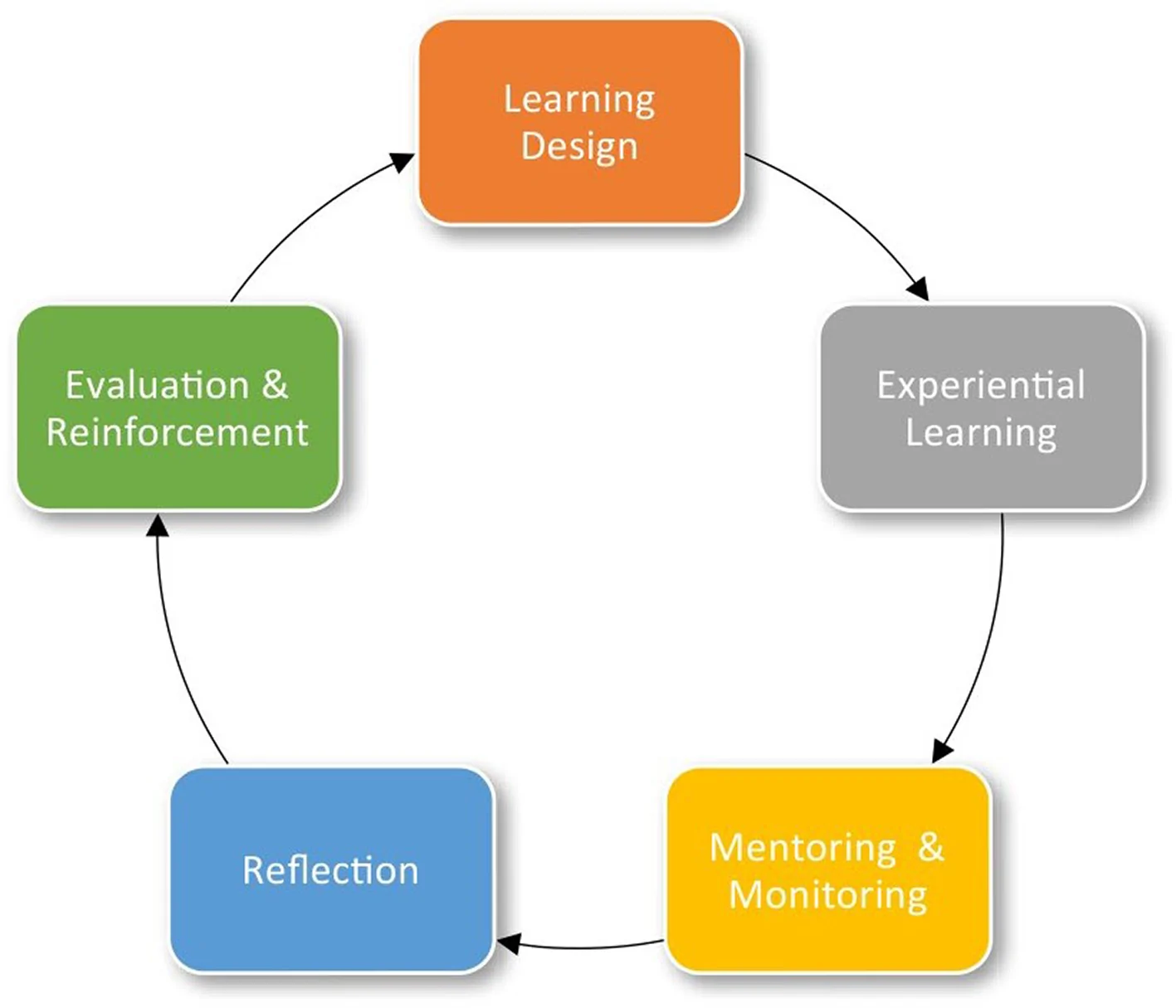
The competence development cycle through learning experiences.

The success of higher education institutions in implementing the independent campus programme depends largely on how well and effectively the programme is managed and implemented. This study found that the development of competencies occurs through a learning process that is gradual, contextual and reflective, with an emphasis on students’ direct engagement in the real world. This process is cyclical, comprising planning, hands-on experience, guidance, reflection and evaluation. Through this process, students not only acquire knowledge but also develop skills, a professional attitude and a more comprehensive readiness to enter the world of work. Figure 1, illustrates the learning cycle developed by the universities studied to enhance their graduates’ competencies.

**Learning Design**. The learning design or learning plan within the independent campus programme is the initial stage that forms the foundation for the direction, quality and success of the entire learning programme. This stage is designed by the university in a systematic and collaborative manner involving students, academic supervisors and university partners to ensure that the activities undertaken are aligned with the expected learning outcomes. This planning involves setting objectives, learning outcomes and the type of programme in consultation with the supervising lecturer.

**Experiential Learning.** Real-world experience lies at the heart of the independent campus programme, which gives students the opportunity to engage directly in practice-based learning activities in the workplace or within the community. At this stage, students learn through hands-on experience (learning by doing), enabling their skills to develop in a more contextual and practical manner. Through direct involvement, interaction and ongoing feedback, students are able to fully integrate their knowledge, skills and professional attitudes. This is what makes the learning experience in the independent campus programme more meaningful and relevant to the needs of the workplace.

**Mentoring dan Monitoring**. Mentoring and monitoring are supporting processes that ensure the independent campus programme runs in a focused, controlled and meaningful manner. This stage involves the university or the supervising lecturer in providing ongoing, reflective and supportive guidance to students. Through structured guidance, monitoring and feedback, students will not only undergo a learning experience, but will also be able to transform it into meaningful and sustainable skills.

**Reflection.** The stages of making sense of learning experiences, which aim to enable students to internalise the knowledge, skills and attitudes acquired during the learning process. Through reflection, the students seek to understand and evaluate the outcomes or impact of the programme, and then strive to continue developing themselves so that the skills they have acquired become more firmly established and sustainable.

**Evaluation and Reinforcement.** The entire process undertaken by students must be reviewed comprehensively to ensure that the learning experience truly yields the expected outcomes. Evaluation and reinforcement serve not only as a final evaluation, but also as a process for confirming, reinforcing and internalising the skills that have been acquired. So that students not only successfully complete this programme, but are also fully prepared to apply what they have learnt in the workplace and in their future career development.

The success of a program is essentially determined by the extent to which its established objectives are achieved. Such achievements do not happen automatically, but rather through a series of planned stages, a systematic implementation process, and appropriate, well-targeted mechanisms. Through this process, the independent campus program is expected to produce graduates who are not only academically outstanding but also possess the competencies, readiness, and competitiveness required by the job market.

The findings of this study also indicate that the independent campus programme will be successful if implemented through a process of student empowerment. In other words, this programme is not merely an academic activity, but a process that places students at the centre of the learning experience.

### 4.2 Independent campus: Empowering student

Research findings indicate that the implementation of the independent campus programme has had a significant empowering effect on students. Respondents view this programme as a form of learning that provides students with ample opportunities to take an active role in managing their own learning process. Students are free to make their own academic choices, such as selecting courses, drawing up their timetables, and choosing learning methods that suit their personal interests and goals. Furthermore, the flexibility offered by this programme is seen as a key strength. This flexibility allows students greater freedom to explore their potential, enrich their learning experience and develop their skills in a context-specific manner. The independent campus programme serves not only as an alternative learning scheme, but also as a means of empowerment that fosters students’ independence, creativity and personal development through direct involvement in real-world learning experiences, thereby ensuring they are better prepared and more mature when facing the demands of the workplace. Some examples of interview results with respondents are as follows.

*“The independent campus programme has given us the freedom to choose our programmes, organise our learning and development … the independent campus programme provides support and opportunities for us to develop our creativity and independence in seeking and discovering new knowledge through field dynamics” (S-1).*

*“Something special for me is carrying out industry practice learning through certified independent study that supports knowledge and work experience in fun learning activities from experts” (S-2).*

*“As a college graduate who has participated in the independent campus programme, of course I really appreciate this programme … provided me with valuable experience, such as machine shop practice and this practice is very supportive of my current profession” (G-1).*

Based on the opinions of the participants above (S-1, S-2 and G-1) that the independent campus programme provides many experiences and benefits for them. They also feel that they are given the opportunity to learn and choose programmes according to their interests and talents so that their skills and experience are developed through field practice. Their learning is guided by lecturers and experts, so they have a broad knowledge and high expectations to succeed in their studies. The findings of this research provide an explanation that they are empowered through the independent campus programme. Empowerment is both a goal and a process to achieve a goal that is to be achieved with strength or power that leads to individual or group independence. The findings of this research have also identified six aspects of empowerment in the independent campus programme in higher education that have an impact on the achievement of higher education goals, including: (1) enabling, (2) facilitating, (3) consulting, (4) collaborating, (5) mentoring, (6) supporting. The following are the themes of the findings based on the results of the research data analysis.

***4.2.1 Enabling***

Universities in running independent campus programmes consider the enabling aspect so that students can take control of their education so that they reach their maximum potential. The process of enabling students with the independent campus programme aims to provide opportunities for students to take part in programmes and choose courses outside the study programme according to their interests and talents. Enabling also encourages students to explore new things, develop themselves according to their career goals and interests. Enabling is not just about providing access, but also about empowering students to play an active role in the learning process and develop potential. One example from the interviews explains:

*“I used to be a shy and passive person, but since being actively involved in the entrepreneurship programme, I have gradually turned into an optimistic person. The journey of this entrepreneurship project is certainly through an intensive mentoring process by lecturers so that I arrive at knowledge about the entrepreneurial process, opportunity recognition, business strategies, market opportunities, marketing, business capital and others. The most important experience I gained from the entrepreneurship programme is the ability and confidence in entrepreneurial projects and the desire to succeed in business. Although at the beginning of the implementation, I felt difficult, hesitant and had low confidence in the project, these feelings have now disappeared. Considering that an independent campus has great benefits, it is certainly very much needed by students and not only fosters enthusiasm, but builds thinking concepts and encourages practical entrepreneurial skills in graduates” (S-3).*

Entrepreneurship is one of the independent campus programmes and a tool for students to express and actualise themselves. As students of the independent campus programme, S-3 explained the important aspect that the bar to their success is set by their own creativity, enthusiasm and vision. S-3 is a student of the economics and business study programme, he actively carries out entrepreneurial practices assigned from the campus. The task in her entrepreneurship project is online-based skincare product entrepreneurship. During his entrepreneurial practice, he was guided by a supervisor who had entrepreneurial experience. Supervision is carried out by lecturers until the end of the entrepreneurial project and performance reporting. This entrepreneurship programme from the campus is given to students who have an interest in entrepreneurship to develop their business early and guided. Through coaching from supervisors and assistance from skincare business partners, she succeeded even though product marketing is still within the scope of relatives and close people. S3 explained that the practice of entrepreneurship provides benefits to his development, as an independent, creative and productive figure, dare to take risks and be responsible. The enabling aspect of the independent campus programme is effective in turning students into individuals who are resilient, tough and have a vision to always succeed in business. In addition, they become more creative and innovative in creating business and economic opportunities for themselves and for others.

***4.2.2 Facilitating***

The facilitating aspect of the independent campus programme refers to efforts to provide students with easy access to various learning opportunities outside the standard curriculum. In this programme, universities, through their lecturers, have the responsibility to provide information to students about the programmes available. They also provide guidance and advice to students during the process of independent campus activities. Here is one example of an interview with a lecturer:

*“As lecturers in the independent campus programme, we are the driving force that makes it easy for students to gain skills, knowledge, and experience. We are also required to implement collaborative classes and increase practice, conduct research with students and guide students in learning when the independent campus is implemented”* (L-1).

The role of lecturers in an independent campus is to facilitate students for the smooth implementation of an independent campus. The lecturers explained that they facilitate students in class and outside of class. Outside the classroom, lecturers become student mentors and motivate students to actively participate in various new learning activities in the independent campus programme. When students face difficulties, the lecturer is willing to provide assistance, so that students do not lose their direction and enthusiasm. Lecturers also carry out research activities and involve students or mentor student research. Thus, facilitating in the independent campus programme relates to efforts to create an environment that supports students’ academic and professional development through easier and more flexible access.

***4.2.3 Consulting***

Consulting in the independent campus programme is a process where students consult with lecturers, academic advisors, or other professionals for advice and direction on educational planning. Consultation can also help students identify their competencies and experience. This may include recognition of professional certifications, work experience, or learning that is recognised as part of an independent campus programme. The lecturers argued that apart from being supervisors, they are also consultants who provide the direction needed by students in independent campus activities. They assist students as academic consultants on projects, internships or independent study programmes and more. It can be interpreted that empowerment by consulting helps students ensure that the education they receive is in accordance with their needs and helps them achieve their expected goals. The students also perceived them (lecturers) as facilitators and advisors who support students in their academic journey in the independent campus programme. Help steer students towards success in their education. They not only offer course options or evaluate study plans, but also understand each individual’s background, interests, and goals. They also provide specific and appropriate advice to strengthen students’ academic success.

Mentoring encourages students not only to engage in activities, but also to reflect on their learning experiences so that they can draw meaning and lessons from each activity. Respondents felt that “support and validation from supervisors help students feel more confident in making decisions, expressing ideas and tackling challenges in real-world settings”. A key aspect of this consultation is ensuring that students’ learning experiences are not only practical, but also focused, meaningful and contribute to the optimal development of their skills.

***4.2.4 Collaborating***

One important aspect of a successful independent campus is collaboration. Collaboration in the independent campus programme is a collaboration between students, lecturers, and related parties, such as companies, partners or community organisations to design and implement the programme. Collaborating gives students the opportunity to be actively involved in real-world projects. The respondents explained that collaboration is the most important thing in carrying out various activities on the independent campus. Forms of collaboration between lecturers and students include co-operation in research and field projects. Where lecturers become project mentors, provide methodological guidance, and share insights based on their experience with students. Here are some results of interviews with respondents:

*“Collaboration between lecturers and students is key to creating meaningful learning experiences and building the foundation for student academic success. In independent campus programmes this relationship is not limited to the transmission of knowledge, but also involves mentorship, guidance and support in the personal and professional development of students” (L-4).*

*“Collaborating with lecturers on independent learning projects has really helped us open doors for deeper and more relevant learning. We can learn not only from books and lectures, but also from the practical experiences that lecturers share. In addition, good co-operative relationships with lecturers build confidence and increase our motivation in achieving academic goals” (S-5).*

Based on the explanations of L-4 and S-5, collaboration is an important element in the independent campus programme because it can help connect education with life. Collaboration between lecturers and students can create a meaningful learning experience and build the foundation for a successful programme. According to them, collaboration is not only limited to the transmission of knowledge, but also involves mentorship, guidance, and support in students’ personal and professional development.

***4.2.5 Mentoring***

The presence of lecturers in the independent campus programme is as mentoring students. They guide, provide direction and valuable input on how to deepen academic understanding, and develop critical skills. In addition, the lecturers design programmes that meet the needs of the students and assist the students during their programmes. Some students (such as S-6, S-7 and S-8) thought that they were given a lot of mentoring and practical training in field projects or mentoring in challenging assignments. They also provide feedback to students to reflect on their skills and work. In addition, they are not only in charge of delivering lecture material, but also play a role in directing, motivating, and providing support so that students can develop their potential optimally.

It can be explained that in the implementation of the independent campus, the supervisors have acted as mentors. This empowerment aspect makes students feel supported and guided during the programme, thus increasing the overall effectiveness of the independent campus programme.

***4.2.6 Supporting***

Supporting in the independent campus programme refers to providing various types of assistance and guidance to students to help them overcome challenges and achieve their educational goals. Support on an independent campus is not only limited to academics, but also includes aspects of student life as a whole. Through various types of support, the independent campus programme aims to create an inclusive and supportive educational environment for all students, so that they can develop personally, academically and professionally to their full potential. In this case, the form of support provided can be financial, better access to educational resources, emotional support, training and workshops to develop skills, and other support that can encourage the effectiveness of the independent campus programme. Here are some examples of interview results:

*“The campus supports us by providing assistance and giving us permission to take part in internships and studies at the independent campus programme…” (S-9).*

*“Mentoring support to prepare students to implement the programme to equip students with strengthening leadership skills. We also expand the co-operation system, develop and create strategies to ensure the expected plans and goals can be achieved. Other support provided by the campus is strengthening the role of lecturers in tasks, teamwork, expanding and building collaborative networks with partners” (L-4).*

Empowerment in the independent campus programme is very important. As S-9 explained, support, guidance and direction from the campus are very meaningful for the success of students in implementing the independent campus programme. Empowerment that gives students the opportunity to learn according to their own learning style and encourages them to take responsibility for their learning process. Likewise, L-4 argues that as a lecturer he also acts as a mentor who seeks to help and support students to achieve maximum results in the independent campus programme so as to produce graduates who are superior and have personality. Table 2 summarises the findings of the interview data analysis related to “empowerment” in the independent campus programme and its impact.

**Table 2.** Summary of findings on student empowerment and its impact.

Source of information	Process of empowerment	Theme	Impact
S-1, S-2, S-3, S-4, and S-5; G-1 and L-1, L-2, L-3, L4 and L-5	Encourage students to be capable, provide opportunities, provide access, develop and strengthen the potential of students.	Enabling	Students explore new things; develop themselves and their potential according to their career goals and interests.
S-1, S-3, S-4, S-5, S-6, S-7, and S-9; G-1, G-2 and L-1, L-2, L-3, L-4 and -L5	Provide convenience, facilitate, support the smooth running of student education and develop students’ interests, talents and strengths.	Facilitating	Facilitate programme success and effectiveness; develop students academically and professionally.
S-1, S-2, S-3, S-5, S-6, and S-9; L-1, L-2 and L-3	Providing assistance, positive communication, consultation on success, building students’ ideas, thoughts and potential, solving problems, making decisions, planning studies, determining success and determining students’ careers.	Conculting	Programmes and education received, selected and applied are appropriate to student needs, accelerating student success and goal achievement.
S-1, S-4, S-5, S-6, S-7, S-8 and S-9: G-1, G-2 and L-1, L-2, L-3, L-4 and L-5	Build and develop cooperation between campuses, lecturers, students and partners, teamwork, encourage student involvement with lecturers and partners in independent campus projects.	Collaborating	Encouraging student involvement in activities and projects with lecturers, experts and experts; student involvement in community development; building leadership character, mutual cooperation, co-operation and team skills.
S-2, S-3, S-5, S-6, S-7, S-8 and S-9; L-1, L-2, L-3, L-4, and L-5	Directing, guiding, coaching and training students in independent campus projects; as well as providing direction on student success.	Mentoring	Streamline and accelerate student success in learning and performance of independent campus projects.
S-1, S-2, S-3, S-4, S-5, S-7, S-8 and S-9; L-1, L-2, L-3 and L-4	Facilitate students in developing their interests, talents and potentials; provide various types of assistance, facilities and infrastructure, financial and moral support.	Supporting	Creating an inclusive learning environment for students so that students can develop personally, academically and professionally.

Based on Table 2, the findings of this study show six aspects of empowerment implemented by universities in implementing an independent campus, namely (1) enabling, (2) facilitating, (3) consulting, (4) collaborating, (5) mentoring, (6) supporting. These six aspects of empowerment greatly influence and play an important role in the success of students in implementing independent campus programmes. Through these six forms of empowerment, students are effectively encouraged to become independent, adaptable, reflective learners, enabling them to develop critical thinking skills, collaborate effectively and build self-confidence. The learning experience, built through enabling, facilitating, consulting, collaborating, mentoring and supporting, has helped students become more independent in managing their own learning process, whilst enhancing their critical thinking, communication and collaboration skills. Ultimately, this empowerment helps students develop skills relevant to the needs of the workplace and prepares them to face future professional challenges.

### 4.3 Independent campus: Developing students’ abilities and skills

The independent campus programme is essentially designed to equip students with cross-disciplinary skills relevant to the needs of the workplace. Through this programme, students are expected to have the opportunity to explore the learning process in a flexible manner, thereby gaining the freedom to determine the direction of their studies, develop their interests, and hone skills that align with their individual potential.

The usefulness of the independent campus programme in developing abilities and skills is strongly supported by interview data with research respondents. The respondents (students and graduates) explained that during the independent campus programme, they received many benefits from the programme. Through an independent campus, new experiences are discovered, abilities and skills are developed and can even experience the world of work that is their provision after graduating from college. Here are examples of some of the interview results:

*“The independent campus programme provides many benefits for me as a college graduate…the certified internship programme that I have participated in the past, really supports my current work…such as problem solving skills, analysis, and technical skills” (G-2).*

*“An independent campus is an essential embodiment of learning. Providing challenges and opportunities for me as a student to develop my creativity and capacity to seek and find knowledge through the realities and dynamics of the workplace…the school teaching programme I took was very beneficial to my skills in teaching and leading learning” (S-5).*

Respondents explained that the independent campus programme was effective in developing their skills and abilities. Abilities and skills that support them as they enter the workforce. G-2 is a graduate who explained his experience of joining a certified internship programme at a digital company while he was still at university. She is now working for a digital company and the skills gained from the certified internship programme are supporting her current job. S-5 also believes that the school-based teaching practice programme that she implemented benefited her in developing her teaching and learning leadership skills. The implementation of an independent campus towards student development was also explained by lecturer respondents. One example of an interview is as follows:

*“In carrying out projects with students, as a supervisor, I always encourage them to be more creative and productive in their studies … for example in the village development project, students are invited to actively contribute to the community through this programme. It not only hones soft skills, but also builds cross-disciplinary collaboration and leadership in rural development programmes” (L-3).*

A independent campus programme in higher education is considered effective if it is able to provide context-appropriate practical experience that enhances students’ overall competencies and skills (Summary of research findings as shown in Table 3). The findings of this study have identified eight abilities and skills gained from the independent campus programme, namely: (1) leadership, (2) teaching skills, (3) communication skills, (4) problem solving, (5) critical thinking, (6) analytical skills, (7) networking, (8) technical skill.

**Table 3.** Summary of findings on competence, skills and impact.

Source of information	Programme and characteristics	Theme	Impact
S-4 and S-5	Campus teaching: provide opportunities for students to learn and develop themselves through activities outside the lecture class, improving literacy and numeracy, and technology adaptation in schools, assist learning and improve the quality of learning in schools	Skill Teaching and Learning, Leadership, and Collaboration	Able and skilled to teach very specifically and lead learning
S-1, S-3 and G-1, and L-1	Student Exchange: take classes or semesters at overseas or domestic universities, based on a co-operation agreement. Domestic universities, based on the cooperation agreement that has been held government. Entrepreneurship: developing innovation and creativity in creating something different as well as having the value and ability to face life’s challenges by seeing opportunities from various risks and uncertainties in order to achieve profit and growth. Certified internship: develop direct work experience in the industry or professional world. Improve hard skills, as well as soft skills that are crucial in preparing for the world of work	Communication Skill, Networking, Leadership, Technical Skill	Ability to lead groups and teams Tolerance, respect for cultural differences Building friendships and communicating Increase global career opportunities Developing creativity and innovation
S-6, S-7, S8 and S-9, G3, and L-2	Independent Project: develop a project based on the topic. Research: academic research activities, both science and social humanities, that are conducted under the supervision of a lecturer or researcher	Crtical Thinking, Problem Solving, Analytical Skill	Enhance analytical, critical thinking, problem-solving and industrial talents. Developing creativity and innovation
S-2, G2, and L-3	Certified internship: develop direct work experience in the industry or professional world. Improve hard skills, as well as soft skills that are crucial in preparing for the world of work	Communication Skill, Networking, Technical Skill	Developing creativity and innovation High capability and work ethic Professional expertise

As shown in Table 3, the independent campus programme makes a significant contribution to developing the skills of students and graduates to prepare them for entry into the world of work. Through a flexible, experience-based learning approach, students not only strengthen their academic knowledge but also develop practical skills relevant to the needs of industry. Direct involvement in a range of activities such as work placements, projects, independent study and cross-sector collaboration enables students to hone their technical skills within their academic discipline whilst strengthening soft skills such as communication, collaboration, leadership, critical thinking and adaptability. Furthermore, interaction with the professional environment helps students understand the dynamics of the workplace, organisational culture and real-world performance expectations.

The impact is felt not only during the learning process, but also affects the quality of graduates. The impact on graduates is that they tend to be better prepared to enter the world of work, have practical experience, and are able to adapt to the changes and complexities of the professional world. It can be said that this programme plays a strategic role in producing graduates who are competent, competitive and well-suited to the needs of the labour market.

### 4.4 Relationship patterns

**Figure 2.**
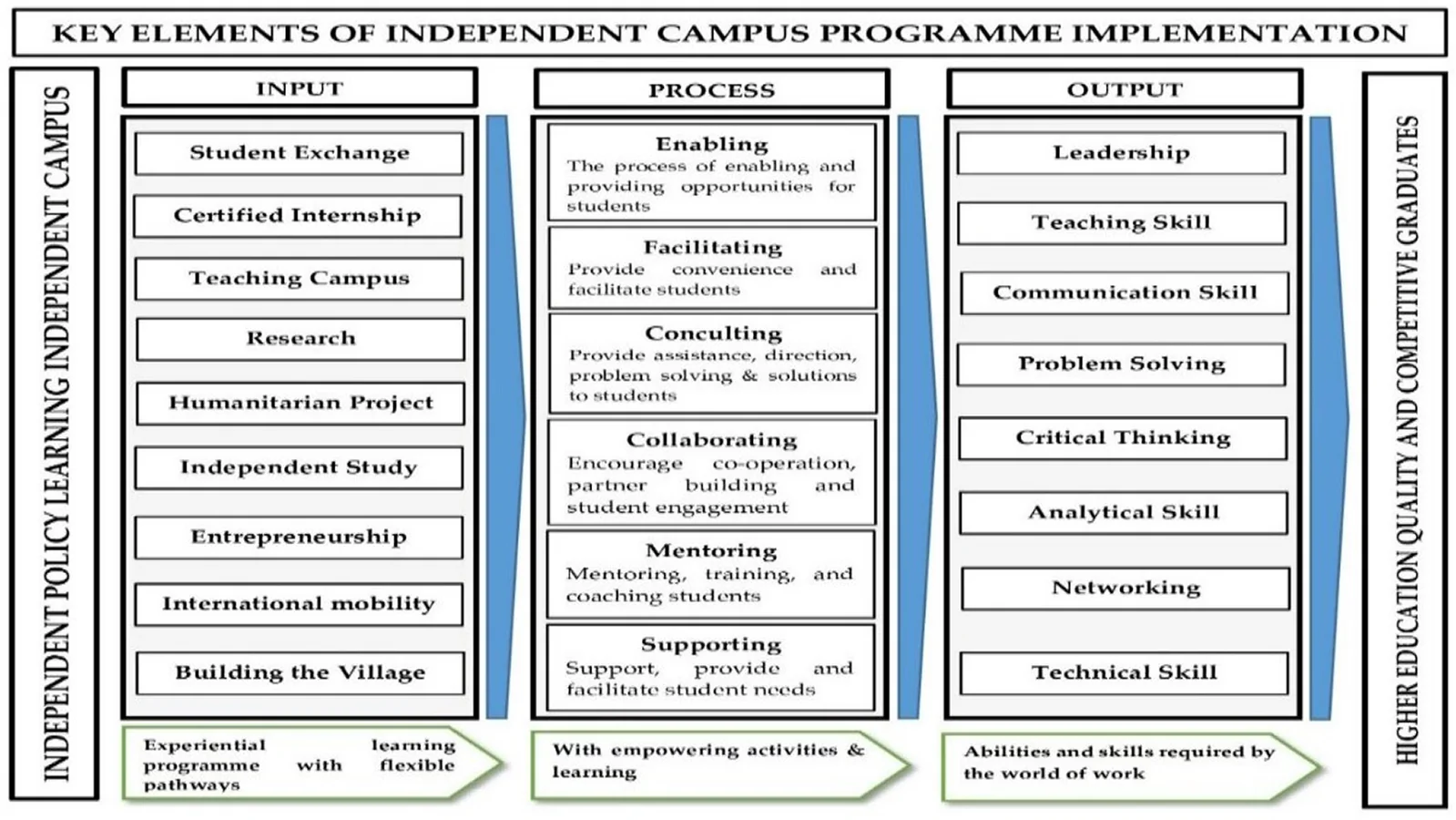
The relationship between the independent campus programme, student empowerment and graduate competencies.

The relationship between the independent campus programme, student empowerment and the development of graduate competencies demonstrates a systematic, causal and sustainable interconnection. The independent campus programme serves as both a policy framework and a learning environment designed to provide flexibility, access to real-world experiences, and opportunities for students to participate actively in the learning process. Through these characteristic, the programme directly fosters the empowerment of students. Student empowerment is reflected in increased independence, decision-making skills, and active involvement in designing and implementing the learning process. Students are no longer seen as passive recipients, but rather as active participants who have control over their own learning process. This process is reinforced through various forms of empowerment support, such as enabling, facilitating, consulting, collaborating, mentoring and supporting. Figure 2 the relationship between the independent campus programme, student empowerment and the development of graduate competencies.

Figure 2 illustrates the relationship between the components of the independent campus programme, student empowerment and graduate competencies. Student empowerment is a key mechanism that transforms learning experiences into tangible skills. Through this process, students are able to develop not only academic skills (hard skills) but also non-academic skills (soft skills) such as communication, teamwork, critical thinking and problem-solving. Thus, the relationship between these three variables can be understood as an integrated process. In other words, the independent campus programme serves as a catalyst for student empowerment, which ultimately leads to the development of graduates’ competencies. From this perspective, student empowerment acts as a mediating variable that is crucial to the programme’s success in producing graduates who are competent, adaptable and ready to meet the demands of the workplace.

## 5. Discussion

The findings of this study identify five stages of the implementation of an independent campus by universities, namely the preparation, implementation, evaluation or assessment, output and outcome stages. In the planning aspect, universities develop policies and guidelines that govern programme implementation. The policy aims to encourage students to master various fields of knowledge in accordance with their fields of interest, namely as a provision and preparation for them to have competitiveness and competitiveness in the global world. Universities also design an independent learning curriculum that gives students flexibility in choosing courses, programmes of study and learning pathways that suit their interests and career goals. This is in line with previous research which shows that curriculum management based on democratisation of education is effective in increasing the active participation of all stakeholders in the planning, implementation and evaluation processes. ^22^ In implementing an independent campus, universities implement project-based learning that encourages students to engage in real-world experiences outside of campus, such as internships, research, or community service. ^10^ In the evaluation stage, the university continuously evaluates the implementation of the programme. In the current era of globalisation, universities are faced with demands for innovation in education and effective management in an effort to achieve superior and globally competitive human resources. ^40, 59^

This study also found that the independent campus programme is an innovative programme that empowers and effectively develops student competencies. Student empowerment is an effort to activate students’ potential and creativity and provide opportunities for them to develop themselves, contribute to society, and achieve academic and career success. As the research findings, empowerment in the independent campus programme in higher education is carried out through a process of (1) enabling, (2) facilitating, (3) consulting, (4) collaborating, (5) mentoring (6) supporting. This student empowerment is done through learning by providing a relevant, innovative, and interactive curriculum that can enhance academic skills, employability skills, and understanding of social, economic, and environmental issues. Student empowerment can be done through internships and practices that provide opportunities for students to take part in internships or work practices in industries or organisations related to their field of study. This helps them gain real-world experience, develop practical skills and expand their professional network. ^16, 17, 53^

The independent campus programme is a learning programme that gives students the right to choose learning that supports their additional abilities and skills in the campus area according to their interests and talents. Through autonomous and flexible learning, a creative and innovative learning culture will be created, accommodating programme choices according to interests and talents so that it is wide open for students to enrich and improve their insights and competencies. ^31^ Independent campuses provide opportunities for students to gain broader learning experiences and new and developing competencies according to the needs of the times. ^33^ Education by giving independence to students will encourage students to be directly involved in the learning process and transfer of knowledge. ^3^ The independent campus programme also supports students to deepen the knowledge of the study programme, can find out things that are not yet known in their own campus, improve the soft skills expected from their own study programme. ^10^

The independent campus programme in higher education is considered effective when the programme objectives can be achieved as expected. The effectiveness of the independent campus programme in higher education is the achievement of the abilities and skills of the students and the competitiveness of their graduates. Higher education programmes are expected to improve the character and abilities of strong and better learners. ^11^ Independent campuses provide contextualised field opportunities that can strengthen students’ overall capabilities, prepare them for employment, or build new careers. ^29, 32^ As the findings of this study show, the independent campus programme is effective in developing students’ abilities and skills. The abilities and skills developed include (1) leadership, (2) teaching skill, (3) communication skill, (4) problem solving, (5) critical thinking, (6) analitycal skill, (7) networking serta (8) technical skill. Programmes that provide practical experience such as internships and field projects tend to improve critical thinking skills and technical skills needed in the world of work. ^12^ This indicates that the independent campus approach that provides freedom of learning and hands-on practice has a strong influence in achieving the targets of university performance indicators such as higher graduate competence and increased graduate competitiveness. ^36, 44^ Students who engage in practical and project-based activities tend to have higher levels of employability compared to students who follow a traditional curriculum. ^5^ The independent campus contributes highly to the main performance indicators of higher education, this is because the independent campus programme is designed to equip students with practical skills that are relevant to industry needs. ^6^ Students who got involved in one or more out-of-class activity on campus acquire specific competencies such as networking, collaboration, communication, leadership, multidisciplinary thinking, etc. ^69^

Noting that the independent campus programme in higher education has a significant contribution to the achievement of the profile of its graduates, the existence of the independent campus programme needs to be maintained and improved in the midst of the demands for quality education in Indonesia. The benefits of the independent campus programme for students are not only helping students develop a democratic culture in campus life, but more than that, namely the development of the ability to build freedom of thought that is not only limited to the lecture environment. ^31^ An independent campus is able to hone the hard and soft skills of students so that in the future they can develop into university graduates with relevant abilities according to the needs of the times. ^42^ High competition in the world of work requires people to step out of their comfort zone. This will only happen if they have trained skills. ^70^

The independent campus programme is a much-needed step forward in Indonesia’s higher education system. However, without adequate preparedness on the part of the campus, the programme risks becoming an ambitious project that fails to achieve its goals. ^16^ With the right corrective measures, independent campuses can be an effective instrument in creating graduates who are not only academically competent, but also have skills and experience that are relevant to the evolving needs of the world of work. ^4, 21^ The realisation of an independent campus as an innovative higher education solution depends on the sincerity of universities in facing challenges and seizing opportunities. ^5^ If managed well, an independent campus will not only produce graduates who are competent and ready to compete in the world of work, but will also form a generation that is creative, innovative, and able to contribute to the progress of the nation. ^25^

## 6. Conclusions

This study concludes that the independent campus programme plays an important role in encouraging student empowerment and contributes to the development of graduate competencies in higher education. Through various forms of flexible learning activities that are relevant to the world of work, such as internships, research, social projects, entrepreneurship, and collaboration with industry, students have more opportunities to actively engage in the learning process. This involvement encourages students to develop independent learning skills, increase their sense of responsibility for their academic progress, and broaden their professional knowledge and experience. Six aspects of empowerment in independent campus programmes at higher education that have an impact on student competency development, namely: (1) enabling, (2) facilitating, (3) consulting, (4) collaborating, (5) mentoring, (6) supporting. Empowering students through the independent campus programme also strengthens various important competencies needed in students’ professional lives, such as (1) leadership, (2) teaching skills, (3) communication skills, (4) problem solving, (5) critical thinking, (6) analytical skills, (7) networking, (8) technical skills, as well as (9) the ability to adapt to changes in the work environment. Overall, the findings of this study confirm that the independent campus programme can be an innovative strategy for improving the quality of learning in higher education while strengthening graduates’ readiness to face the demands of the world of work. Therefore, the sustainability and optimisation of the implementation of the independent campus programme must continue to be supported through the strengthening of institutional policies, the provision of quality human resources (lecturers), increased collaboration with external partners, and the development of more adaptive learning designs that are oriented towards student empowerment.

## Ethical considerations

This study did not involve minors; it obtained written consent from the participants and ensured that their participation was entirely voluntary, and received approval from the University of Muhammadiyah North Sumatra, as stated in research approval letter No. 204/II. 3-AU/UMSU-LP2M/C/2025, and has received research ethics approval from the Indonesian Association of Research Methodology Lecturers (IRMLA).

## Data Availability

The data from this study are not publicly available due to the need to protect respondent confidentiality; however, the data may be accessed subject to the discretion and approval of the University of Muhammadiyah North Sumatra as the research funding body, and upon receipt of a reasonable request submitted to indraprasetia@umsu.ac.id, provided that approval has been obtained from the University of Muhammadiyah North Sumatra confirming that the data will be used solely for academic research purposes, subject to conditions that ensure the protection of sensitive information. Reposipatory: Student Empowerment and Graduate Competency Development in Higher Education: Evidence from an Independent Campus Programme. DOI: https://doi.org/10.6084/m9.figshare.31926951. ^71^ Data are available under the terms of the Creative Commons Attribution 4.0 International license (CC-BY 4.0). PRASETIA, INDRA (2026). Student Empowerment and Graduate Competency Development in Higher Education: Evidence from an Independent Campus Programme. Figure. https://doi.org/10.6084/m9.figshare.31926951.
