# Supplementary material for: Student Empowerment and Graduate Competency Development in Higher Education: Evidence from an Independent Campus Programme
Source: F1000Res. 2026 Jun 25;15:612. Originally published 2026 Apr 24. [Version 2] doi: 10.12688/f1000research.179643.2 (PMC13550920; doi:10.12688/f1000research.179643.2)
Supplement: Peer review history

# Reviewer report: Reviewer response for version 2

**DOI:** 10.5256/f1000research.203655.r496689

**Published:** 2026-07-15

**Authors:** Iwan Asmadi

**Affiliations:** 1 Management, Universitas Bina Sarana Informatika, Central Jakarta, D.K.I. JAKARTA, Indonesia

This manuscript makes a valuable contribution to the literature on the implementation of the Independent Campus Programme, particularly in explaining how student empowerment contributes to the development of graduate competencies. The revised version has significantly improved the quality of the manuscript, especially in terms of methodology and theoretical consistency. However, several methodological and conceptual issues should still be addressed before the manuscript can be considered fully acceptable without reservations.

 Strengths of the Manuscript

 1. Relevant and Timely Research Topic

 2. Improved Research Methodology

 3. Stronger Literature Review

 4. Well-Organized Findings

 Overall Recommendation

 The revised manuscript satisfactorily addresses many of the reviewers' previous comments, particularly regarding methodological consistency, clarification of the research process, and strengthening of the supporting literature.

 Overall, the manuscript is suitable for publication and provides a meaningful contribution to the understanding of student empowerment and graduate competency development within the Independent Campus Programme.

Is the work clearly and accurately presented and does it cite the current literature?

Yes

If applicable, is the statistical analysis and its interpretation appropriate?

I cannot comment. A qualified statistician is required.

Are all the source data underlying the results available to ensure full reproducibility?

Partly

Is the study design appropriate and is the work technically sound?

Partly

Are the conclusions drawn adequately supported by the results?

Partly

Are sufficient details of methods and analysis provided to allow replication by others?

No

Reviewer Expertise:

Areas of expertise in educational management

I confirm that I have read this submission and believe that I have an appropriate level of expertise to confirm that it is of an acceptable scientific standard.

# Reviewer report: Reviewer response for version 2

**DOI:** 10.5256/f1000research.203655.r498461

**Published:** 2026-07-08

**Authors:** Sri Nurhayati

**Affiliations:** 1 Institut Keguruan dan Ilmu Pendidikan Siliwangi, Cimahi, Indonesia

1. Introduction 

1. The introduction provides a comprehensive overview of the Independent Campus Programme and establishes its educational significance.
2. The literature review is current and identifies important issues related to student participation, empowerment, and graduate employability.
3. The research gap is clearly stated; however, the manuscript could more explicitly emphasize how this study advances existing qualitative research on student empowerment rather than primarily extending previous impact studies.
4. The theoretical positioning of empowerment could be synthesized more clearly before presenting the research objectives.
5. Overall, the introduction provides a solid foundation for the study.

2. Research Objective

1. The research objectives are clearly presented and align well with the identified research gap.
2. The objectives appropriately focus on exploring both student empowerment and graduate competency development.
3. Nevertheless, the manuscript would benefit from explicitly stating the research questions that guided the qualitative inquiry.
4. Clarifying the expected theoretical contribution alongside the practical contribution would further strengthen the study.
5. Overall, the objectives are appropriate and coherent with the research design.

3. Methods 

1. The methodology is clearly described and demonstrates good alignment between the qualitative design, participant selection, and thematic analysis.
2. Participant selection criteria and data collection procedures are sufficiently transparent.
3. Nevertheless, additional information regarding coding procedures, theme refinement, and researcher reflexivity would further enhance methodological rigor.
4. Although triangulation through interviews, observations, and documents is mentioned, the manuscript could explain more explicitly how these sources were integrated during analysis.
5. Overall, the methodological quality is appropriate for qualitative educational research.

4. Results / Findings 

1. The findings are systematically organized and supported by rich participant quotations that effectively illustrate each emerging theme.
2. The six empowerment dimensions and competency development themes are presented clearly and logically.
3. The conceptual figures and summary tables substantially improve the readability of the findings.
4. However, several sections remain somewhat descriptive and could provide deeper interpretation regarding why particular empowerment mechanisms produced specific competency outcomes.
5. Overall, the results offer convincing empirical evidence supporting the study objectives.

5. Discussion 

1. The discussion successfully relates the findings to previous literature and demonstrates consistency with contemporary higher education research.
2. The manuscript appropriately explains how student empowerment contributes to competency development within experiential learning environments.
3. The discussion would be strengthened by providing more critical reflection on findings that extend or differ from previous studies rather than primarily confirming existing knowledge.
4. Additional discussion regarding the transferability of these findings beyond the Indonesian higher education context would also enrich the contribution.
5. Overall, the discussion provides meaningful theoretical and practical insights.

6. Conclusion 

1. The conclusion effectively summarizes the major findings and reinforces the educational importance of student empowerment within the Independent Campus Programme.
2. Practical implications for universities and policymakers are clearly identified.
3. The manuscript would benefit from distinguishing more explicitly between immediate institutional implications and broader theoretical implications.
4. Future research recommendations could also encourage comparative studies across institutions or national contexts to validate the proposed empowerment model.
5. Overall, the conclusion is well written and consistent with the study findings.

Is the work clearly and accurately presented and does it cite the current literature?

Yes

If applicable, is the statistical analysis and its interpretation appropriate?

No

Are all the source data underlying the results available to ensure full reproducibility?

Yes

Is the study design appropriate and is the work technically sound?

Yes

Are the conclusions drawn adequately supported by the results?

Yes

Are sufficient details of methods and analysis provided to allow replication by others?

Partly

Reviewer Expertise:

Educational technology, Community Empowerment, Community Education

I confirm that I have read this submission and believe that I have an appropriate level of expertise to confirm that it is of an acceptable scientific standard.

# Response

**Published:** 2026-07-08

**Authors:** INDRA PRASETIA

**Affiliations:** Postgraduate, Universitas Muhammadiyah Sumatera Utara, Medan, Indonesia

Dear Reviewer,

 We would like to thank Sri Nurhayati from the Institut Keguruan dan Ilmu Pendidikan Siliwangi, Cimahi, Indonesia, for her constructive suggestions and feedback regarding the importance of the theoretical and practical contributions of the research, the integration of data (interview results, observations and documentation), and the need to discuss the research findings within a broader context.

 We are very pleased and grateful for your recommendations regarding our article.

 Best regards

 Indra Prasetia

# Reviewer report: Reviewer response for version 1

**DOI:** 10.5256/f1000research.198177.r479746

**Published:** 2026-05-29

**Authors:** Iwan Asmadi

**Affiliations:** 1 Management, Universitas Bina Sarana Informatika, Central Jakarta, D.K.I. JAKARTA, Indonesia

The research design only mentions a descriptive qualitative study, which is often considered too general and methodologically insufficiently rigorous. It lacks a clear methodological identity. There is no statistical analysis or statistical interpretation, as the study is purely qualitative in nature. Furthermore, the study does not specify its research paradigm. As a result, the epistemological basis underlying the interpretation of the findings remains unclear.

 The sampling strategy is also relatively weak, as the study involved only 17 participants with an unbalanced composition. In addition, there is no explanation regarding data saturation. The authors do not explain how data saturation was achieved, when the interviews were terminated, or what indicators were used to determine saturation.

 The triangulation process is also weak. Although the authors mention interviews, observations, and document reviews, the observation procedures are not clearly described, the types of documents analyzed are not specified, and the process of triangulation itself is not adequately explained. Consequently, triangulation appears to function merely as a methodological formality.

 Overall, the research findings are still acceptable; however, the empirical evidence supporting the findings is not sufficiently strong from an academic perspective. The conclusions drawn are generally reasonable, but they remain overly broad, normative, and extend beyond the actual strength of the available data.

 `

Is the work clearly and accurately presented and does it cite the current literature?

Yes

If applicable, is the statistical analysis and its interpretation appropriate?

I cannot comment. A qualified statistician is required.

Are all the source data underlying the results available to ensure full reproducibility?

Partly

Is the study design appropriate and is the work technically sound?

Partly

Are the conclusions drawn adequately supported by the results?

Partly

Are sufficient details of methods and analysis provided to allow replication by others?

No

Reviewer Expertise:

Areas of expertise in educational management

I confirm that I have read this submission and believe that I have an appropriate level of expertise to confirm that it is of an acceptable scientific standard, however I have significant reservations, as outlined above.

# Reviewer report: Reviewer response for version 1

**DOI:** 10.5256/f1000research.198177.r479747

**Published:** 2026-05-19

**Authors:** Samuel O. Babalola

**Affiliations:** 1 University of Ibadan, Ibadan, Nigeria

This is a very exciting study explaining the importance of co-creation activities on students' job skills development. The authors have provided a great perspective from the Indonesian lens. However, I will suggest that the authors need to provide a stronger justification for carrying out this study, particularly because they cited past studies that have reported the effectiveness of the independent campus programme.

 Furthermore, the authors need to do a thorough editing of the manuscript. In part of the manuscript, offers was written as "of fers" and the word "and" appeared in some places in the manuscript where they were not needed.

 The authors did not reference similar approaches, studies  or efforts that seek to provide a holistic campus experience to students, such as campus co-creation and out-of-class engagement programmes in other places or regions. I have recommended a study that could be cited in this regard.

 The study results were well presented and the discussion was carefully done. The study will make a great addition to existing knowledge in these areas and it also helps to know what is goin on in the Indonesian context. 

 Based on this few comments, I suggest the paper be accepted with minor revison

Is the work clearly and accurately presented and does it cite the current literature?

Yes

If applicable, is the statistical analysis and its interpretation appropriate?

Yes

Are all the source data underlying the results available to ensure full reproducibility?

Yes

Is the study design appropriate and is the work technically sound?

Yes

Are the conclusions drawn adequately supported by the results?

Yes

Are sufficient details of methods and analysis provided to allow replication by others?

Yes

Reviewer Expertise:

Co-creation and out-of-class engagement for job skills development in higher education, Curriculum and instruction delivery and Education for sustainability in higher education.

I confirm that I have read this submission and believe that I have an appropriate level of expertise to confirm that it is of an acceptable scientific standard, however I have significant reservations, as outlined above.

## References

[1] Students-staff classroom co-creation as model of employability skills development in a Nigerian university author.

# Response

**Published:** 2026-06-09

**Authors:** INDRA PRASETIA

**Affiliations:** Postgraduate, Universitas Muhammadiyah Sumatera Utara, Medan, Indonesia

Responses to Peer Review Report of Samuel O. Babalola, Ph.D, University of Ibadan, Ibadan, Nigeria

 Dear Samuel O. Babalola, Ph.D

 We sincerely thank the reviewers for their insightful comments and constructive feedback, which have greatly improved the quality and clarity of this manuscript. Below, we address each point in detail and describe the corresponding revisions made.

**Comment: **This is a very exciting study explaining the importance of co-creation activities on students' job skills development. The authors have provided a great perspective from the Indonesian lens. However, I will suggest that the authors need to provide a stronger justification for carrying out this study, particularly because they cited past studies that have reported the effectiveness of the independent campus programme. Furthermore, the authors need to do a thorough editing of the manuscript. In part of the manuscript, offers was written as "of fers" and the word "and" appeared in some places in the manuscript where they were not needed.The authors did not reference similar approaches, studies  or efforts that seek to provide a holistic campus experience to students, such as campus co-creation and out-of-class engagement programmes in other places or regions. I have recommended a study that could be cited in this regard.

 The study results were well presented and the discussion was carefully done. The study will make a great addition to existing knowledge in these areas and it also helps to know what is goin on in the Indonesian context

**Response**: We have addressed the suggestion to correct the spelling of ‘of fers’ to ‘offers’, as well as the use of ‘and’ in certain parts of the manuscript where it was not strictly necessary.

**Comment :** Reference

**Response:** We have included the suggested references "Students-staff classroom co-creation as model of employability skills development in a Nigerian university" in the manuscript to enrich the information drawn from other studies.
